# Transcriptome analysis of the digestive system of a wood-feeding termite (*Coptotermes formosanus*) revealed a unique mechanism for effective biomass degradation

**DOI:** 10.1186/s13068-018-1015-1

**Published:** 2018-02-03

**Authors:** Alei Geng, Yanbing Cheng, Yongli Wang, Daochen Zhu, Yilin Le, Jian Wu, Rongrong Xie, Joshua S. Yuan, Jianzhong Sun

**Affiliations:** 1 Biofuels Institute of Jiangsu University, School of Environmental and Safety Engineering, Jiangsu University, Zhenjiang, 212013 Jiangsu China; 2 Synthetic and Systems Biology Innovation Hub, Department of Plant Pathology and Microbiology, Texas A&M University, College Station, TX 77843 USA

**Keywords:** Biomass deconstruction, RNA-seq, Glycoside hydrolase, Auxiliary redox enzyme, *Coptotermes formosanus*

## Abstract

**Background:**

Wood-feeding termite, *Coptotermes formosanus* Shiraki, represents a highly efficient system for biomass deconstruction and utilization. However, the detailed mechanisms of lignin modification and carbohydrate degradation in this system are still largely elusive.

**Results:**

In order to reveal the inherent mechanisms for efficient biomass degradation, four different organs (salivary glands, foregut, midgut, and hindgut) within a complete digestive system of a lower termite, *C. formosanus*, were dissected and collected. Comparative transcriptomics was carried out to analyze these organs using high-throughput RNA sequencing. A total of 71,117 unigenes were successfully assembled, and the comparative transcriptome analyses revealed significant differential distributions of GH (glycosyl hydrolase) genes and auxiliary redox enzyme genes in different digestive organs. Among the GH genes in the salivary glands, the most abundant were GH9, GH22, and GH1 genes. The corresponding enzymes may have secreted into the foregut and midgut to initiate the hydrolysis of biomass and to achieve a lignin-carbohydrate co-deconstruction system. As the most diverse GH families, GH7 and GH5 were primarily identified from the symbiotic protists in the hindgut. These enzymes could play a synergistic role with the endogenous enzymes from the host termite for biomass degradation. Moreover, twelve out of fourteen genes coding auxiliary redox enzymes from the host termite origin were induced by the feeding of lignin-rich diets. This indicated that these genes may be involved in lignin component deconstruction with its redox network during biomass pretreatment.

**Conclusion:**

These findings demonstrate that the termite digestive system synergized the hydrolysis and redox reactions in a programmatic process, through different parts of its gut system, to achieve a maximized utilization of carbohydrates. The detailed unique mechanisms identified from the termite digestive system may provide new insights for advanced design of future biorefinery.

**Electronic supplementary material:**

The online version of this article (10.1186/s13068-018-1015-1) contains supplementary material, which is available to authorized users.

## Background

Lignocellulosic biomass utilization represents an essential path for sustainable production of fuels and chemicals towards petroleum displacement. Despite decades of extensive research, efficient degradation and conversion of biomass remain a major challenge for modern biorefinery. In particular, biomass conversion depends heavily on the pretreatment process to deconstruct biomass and to make cellulose and hemicellulose accessible for enzymatic saccharification. The pretreatment process usually demands extreme temperature, pH, and pressure, which will result in high-energy input and capital investment [1]. In this regard, the environmentally friendly and cost-effective strategies for biomass deconstruction are imminently needed, and as a matter of fact, the mechanisms in natural biomass utilization systems (NBUS) could potentially provide invaluable insights into the design of new biorefinery strategies [2].

Various NBUS have evolved to efficiently degrade and utilize natural biomass under amiable temperature, pH, and pressure [2, 3]. Extensive work has gone into understanding the different mechanisms of lignin modification or depolymerization in these model systems. These include wood-rot fungi and wood-feeding termites, where lignin is the main component contributing to biomass recalcitrance [2, 4]. Among all of the NBUS, lower wood-feeding termites (characterized with the symbiotic protists in their gut system) stand out as a unique system to guide the development of biomass processing due to several reasons. First, lower wood-feeding termites represent a very efficient biomass degradation system that accomplishes the degradation processing in hours instead of weeks or months in a fungal system [5, 6]. Second, this type of wood-feeding termite can selectively modify and decompose the lignin by ~ 25%, yet accomplish a maximized utilization of cellulose at > 90% and various hemicellulose components at ~ 60% in their digestive system [6–8]. The mechanism behind the effective biomass deconstruction that enables carbohydrate utilization will be effective for biorefinery design. Third, as a unique biological conversion model, the termite’s digestive gut system can efficiently perform rapid conversion of biomass at room temperature, without a requirement for extreme pH, high pressure, and high temperature as current industry applied. Overall, the termite’s rapid, effective, and ambient environment, deconstruction of biomass, and its efficient mechanism to utilize carbohydrate provided a unique model system for modern biorefinery design [6, 9].

Considering all of the unique advantages mentioned above, extensive research has been carried out to investigate the mechanisms for efficient biomass degradation in the gut systems of these wood-feeding termites. Oxidative modification of lignin has been identified as a major step of biomass deconstruction within the *Coptotermes formosanus* termite, which was in recent confirmed through thermochemolysis and pyrolysis gas chromatography/mass spectrometry [10, 11]. The similar mechanisms were also found in a *Zootermopsis angusticollis* termite and an *Anoplophora glabripennis* beetle [12]. However, the key auxiliary redox enzymes [13] involved in the oxidative modification process are still yet to be found systematically. Even though some enzyme studies mainly focus on laccase and peroxidase for lignin degradation [14, 15], most of the “omics” works only focus on the glycosyl hydrolases involved in cellulolytic functions, despite the importance of the auxiliary redox enzymes involved in lignin modification [16–19].

In addition, the synergistic processing effect between host termite and its gut symbionts also contribute to the highly efficient biomass deconstruction. The digestive system of *C. formosanus* is characterized as a model dual system [20], where the host termite and its gut symbiotic microbes, such as protists and bacteria, synergize the degradation of biomass. Tartar et al. [21] fractionated the host and symbiont microbes, and revealed that each fraction contained a series of cellulolytic genes in the transcriptome analyses. Despite the progress being made, due to the challenges to obtain the complete parts of a termite digestive gut system, few studies were able to successfully investigate different parts of a termite gut system (e.g., salivary glands, foregut, midgut, and hindgut) individually. An in-depth “omics” analysis of different organs in the termite gut digestive system would reveal the biocatalytic network in each part and, reveal the detailed mechanisms for biomass deconstruction.

In this study, the digestive system of the wood-feeding termite, *C. formosanus,* was dissected and divided into four successive parts: salivary glands (SG), foregut (FG), midgut (MG) and hindgut (HG) for total RNA isolation, and high-throughput RNA sequencing. Transcriptional profiling revealed the coordination of carbohydrate-active enzymes and auxiliary redox enzymes in different parts of termite gut systems. The synergy between termite host and its eukaryotic symbionts was analyzed and discussed. RT-PCR analysis was further carried out to analyze the responses of key auxiliary redox enzymes to the diet containing higher lignin contents. The study revealed that termite digestive system synergized the hydrolysis and redox reactions in a programmatic processing through different parts of its gut system to achieve maximized deconstruction and utilization of biomass.

## Results

Previous studies indicate that lignin degradation processing might primarily occur in the foregut and midgut of *C. formosanus* [22]. The ‘pretreated’ biomass will then be further degraded and utilized in the hindgut, where symbiotic protists, with other associated microbes, would play an inevitable role in accomplishing the carbohydrate utilization. In this study, we mainly focused on the transcriptome analysis of four successive digestive organs of *C. formosanus*, including the SG, FG, MG, and HG (Additional file 1: Figure S1), as well as some potential interactions between the termite host and its symbiotic protists residing and functioning in the hindgut.

### Transcriptome sequencing and assembly

An average of 14.3 million paired-end reads, with length of 90 bp, was obtained from the four libraries: the SG, FG, MG, and HG libraries. After assembling these reads into unigenes and discarding the unigenes shorter than 200 nt, overall, 71,117 unigenes were obtained. These ranged in length from 200 nt to 17,769 nt, with an N50 unigenes length of 648 nt (Additional file 2: Table S1). The RNA sequencing contains primarily the eukaryote RNA, covering both termite host and gut protist sequences. The coverage was much deeper than existing studies and thus allowed us to obtain detailed insights for biomass degradation [18, 19, 21].

### Taxonomic analysis of assembled unigenes

**Fig. 1:**
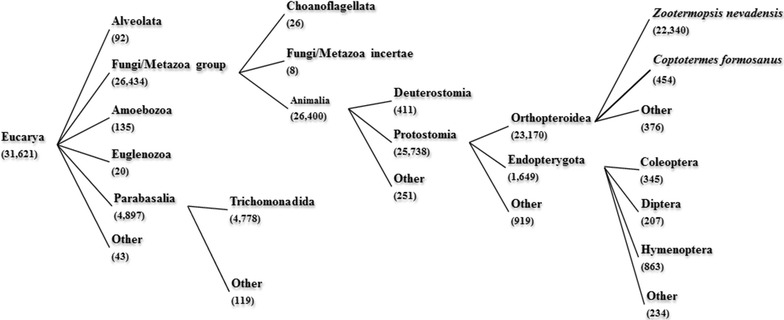
Taxonomy tree of unigenes. The hierarchical structure of the organism classification is based on the NCBI Taxonomy database. The number under the taxa name is the number of unigenes assigned to the corresponding classification from the four termite digestive tissue: *SG* salivary gland; *FG* foregut; *MG* midgut; *GH* hindgut

The assembled unigenes were annotated by searching against the NCBI NR database, using BLASTx with a cutoff *E*-value of 1E−5. Of 31,621 (44.5%) unigenes presented, at least one significant hit was found in the NR database (Additional file 3: Table S2). Based on the taxonomic annotation and the organism classification of the NCBI taxonomy database, a hierarchical taxonomy tree was constructed for the unigenes (Fig. 1). Two major groups in this tree were the Fungi/Metazoa group and the Parabasalia group. Most of the Fungi/Metazoa group was composed of Endopterygota, insects of the subclass Pterygota. Parabasalia are a group of anaerobic protists, some of which are insect symbionts [23]. The number of unigenes assigned to the Fungi/Metazoa group was 26,434, which accounted for 83.6% of all the annotated unigenes, and was nearly 5 times as large as that of the Parabasalia group. Overall, the diversity of unigenes from insects dominated that of the symbiotic protists which resided in hindgut.

As shown in Additional file 4: Figure S2, a higher percentage of unigenes was classified as protistan genes in HG, as compared to SG, FG, and MG. This small quantity of protists in the SG, FG, and MG could be due to a certain degree of contamination during dissection. Due to the technical focus, prokaryote transcripts were not recovered from the samples, although prokaryotes indeed contribute to biomass degradation in termite guts [24, 25]. The taxonomy analysis further imposed an intriguing scientific question regarding how insect host and the protists function coordinately to accomplish the biomass degradation process.

### Gene ontology annotation of unigenes

**Fig. 2:**
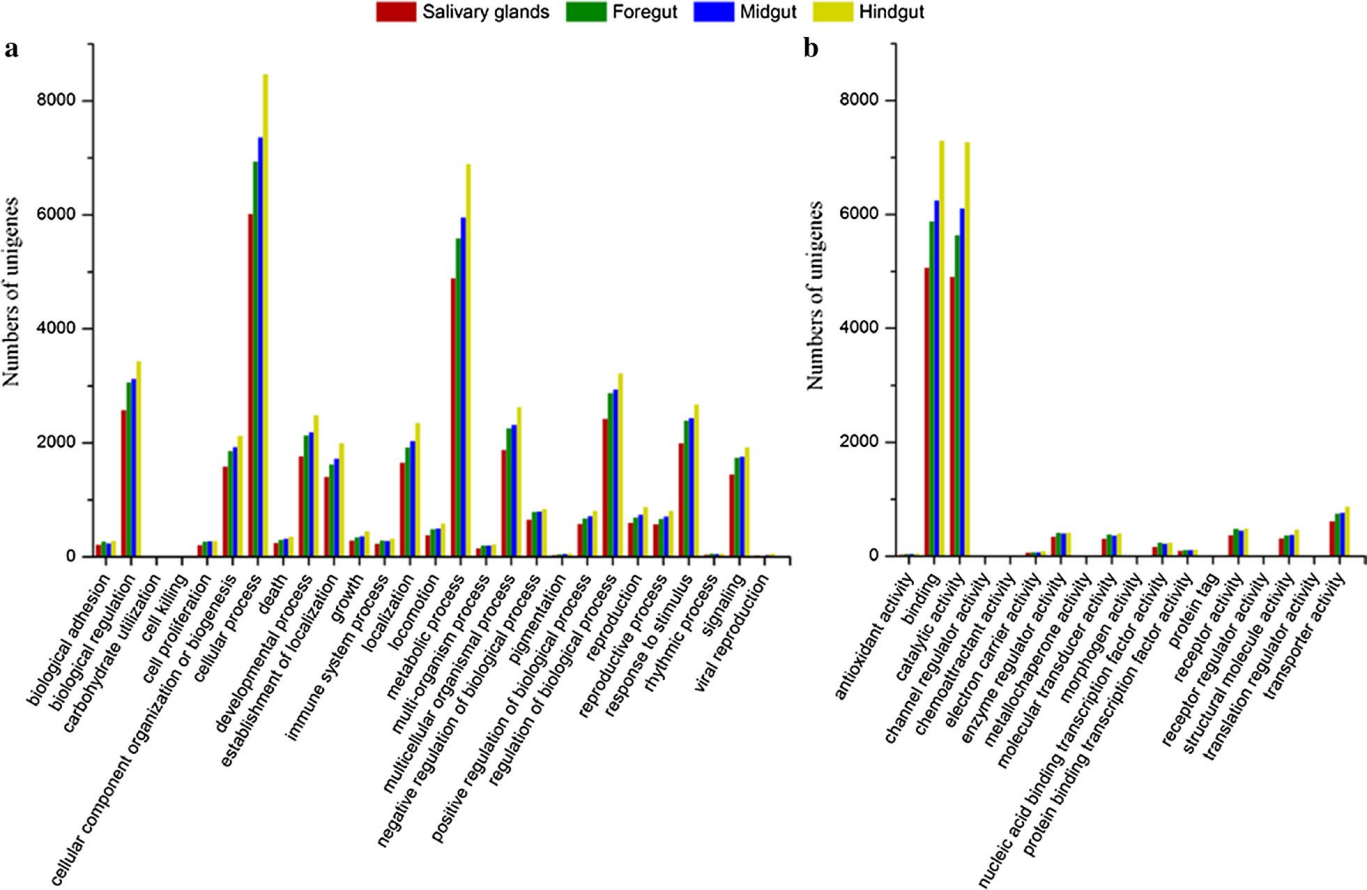
Gene ontology (GO) distribution of unigenes under the category of **a** Biological process and **b** Molecular function

Gene ontology (GO) analysis was carried out to analyze the function distribution of unigenes. About 14,698 unigenes (20.7%) could be assigned to specific GO slim categories. Figure 2 presents the number of unigenes in each GO slim under the category of biological process and molecular function in the SG, FG, MG, and HG tissues. For the biological process category of GO, the two dominant terms were “cellular process” and “metabolic process.” For the molecular function category of GO, the dominant terms were “catalytic activity” and “binding.” The overall distribution patterns of GO terms in the SG, FG, MG, and HG were very similar. The results highlighted biocatalysis as a major function of termite gut.

### Identification of carbohydrate-active enzymes

**Fig. 3:**
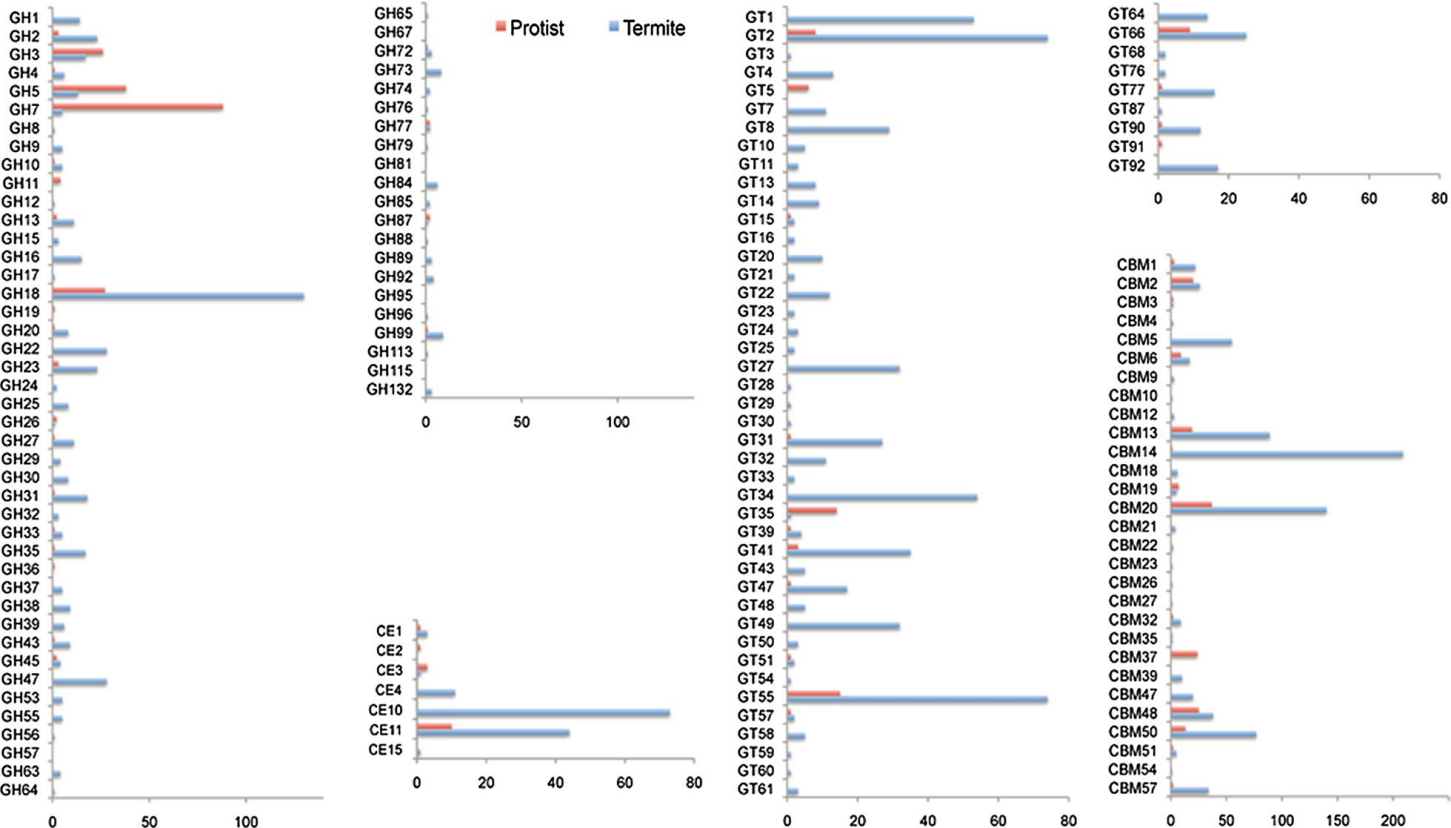
Distribution of identified CAZyme genes encoding for *GH* Glycoside Hydrolases; *GT* Glycosyl Transferases; *CE* Carbohydrate Esterases; *CBM* Carbohydrate binding modules. Unigene that cannot be assigned as termite originated or protists originated are not shown

A comprehensive analysis of CAZymes revealed coordination of the termite host and the symbiotic protists for biomass degradation. CAZymes were responsible for the deconstruction of carbohydrates from biomass in biological systems. As shown in Fig. 3, Additional file 5: Figure S3, over 65% of the glycosyl hydrolases (GHs), glycosyltransferases (GTs), carbohydrate esterases (CEs), and carbohydrate binding domains (CBMs) were from the host species, while about 10 to 27% of those were from the protists. There were also eight unigenes of polysaccharide lyases (PLs), out of which only two unigenes could be assigned to a taxonomy origin, both from termites. Therefore, for the aforementioned five categories of CAZymes, the host has more diverse enzymes than the symbiotic. However, the situation for specific enzyme families might be bit different. For GH7 and GH5, protists actually owned more diverse enzymes than the host (Additional file 5: Figure S3). GH7 and GH5 from protists were actually the largest two GH families involved in biomass degradation with cellulolytic activities.

For CEs, CBMs, and GTs, the differential distribution between the host and symbionts was also obvious. CE11 was the largest CE family, yet the enzymes were usually *N*-acetylglucosamine deacetylases. Therefore, the two largest CE families directly relevant to biomass degradation were actually CE10 and CE4, both of which were exclusively found from the termite host. Similarly, the largest CBM family, CBM14 was mainly for the chitinase. CMB20 and CBM13 represented two major CBM families involved in biomass degradation. Both gene families existed in both the host and the protists. GT55 and GT2 were the top two families of GTs, which also existed in both termite host and its gut protists, but were dominated by the termite host.

### Expression profile of carbohydrate-active enzymes

**Fig. 4:**
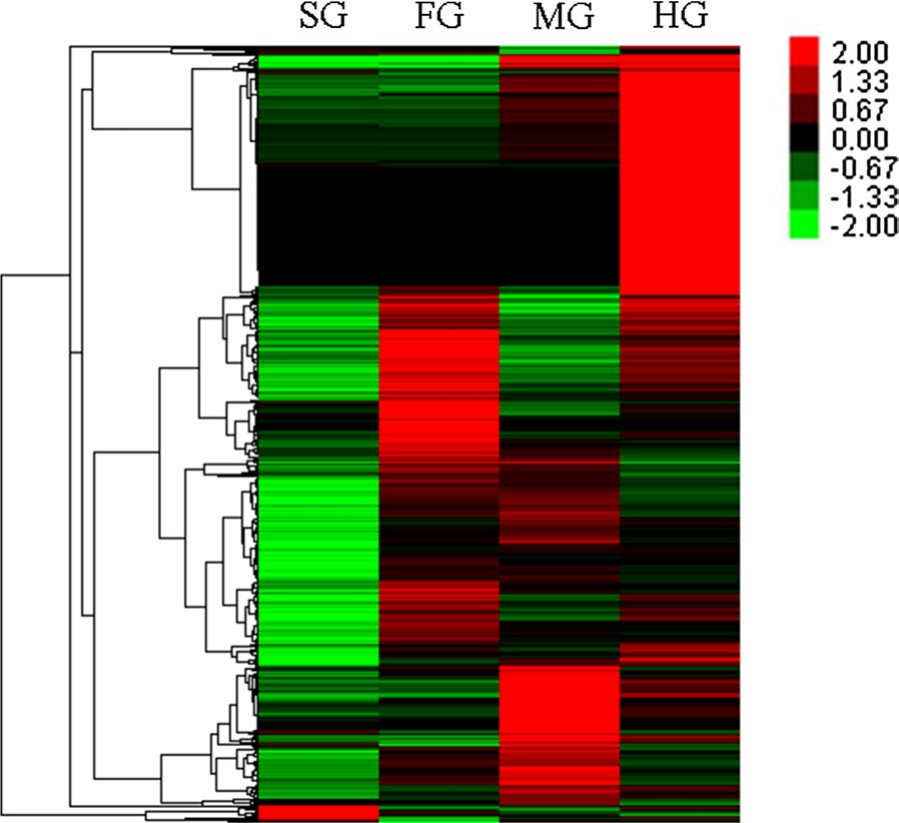
Hierarchical clustering of CAZymes gene expression. Each column represents one sample and each row represents one CAZymes gene. Expression value was normalized in each gene row

A cluster analysis of the transcripts of carbohydrate-active enzymes revealed that most of the genes were specifically transcribed in different parts of the gut system, suggesting the unique functions may be present in each part (Fig. 4). Specifically for the GHs, among the 91 most highly expressed GH genes (FPKM (fragments per kilobase of transcript, per million fragments sequenced) > 100, account for 93% of total FPKMs of GHs from Additional file 6: Table S3), 18, 9, 22, and 42 (26 from protists) unigenes were highly expressed in SG, FG, MG, and HG, respectively, implying that all the four parts of gut tissue might play indispensable roles in biomass hydrolysis. The top 2 GH transcripts among all transcripts (FPKM > 75,000, *CL996.Contig1_All* and *Unigene11767_All*), belonged to GH9 and were annotated from the SG of termite.

Out of all the protistan transcripts, a GH7 cellulase (*CL1544.Contig1_All*), from *Pseudotrichonympha grassii*, was the most abundant (FPKM value = 6737), followed by a GH11 (CL1201.Contig2_All from *Holomastigotoides mirabile*) and another GH7 gene (CL1341.Contig1_All from *H. mirabile*). The transcript profiling further verified the dominancy of GH7 enzymes in protists.

### Termite host encoded most of the AA enzymes

**Fig. 5:**
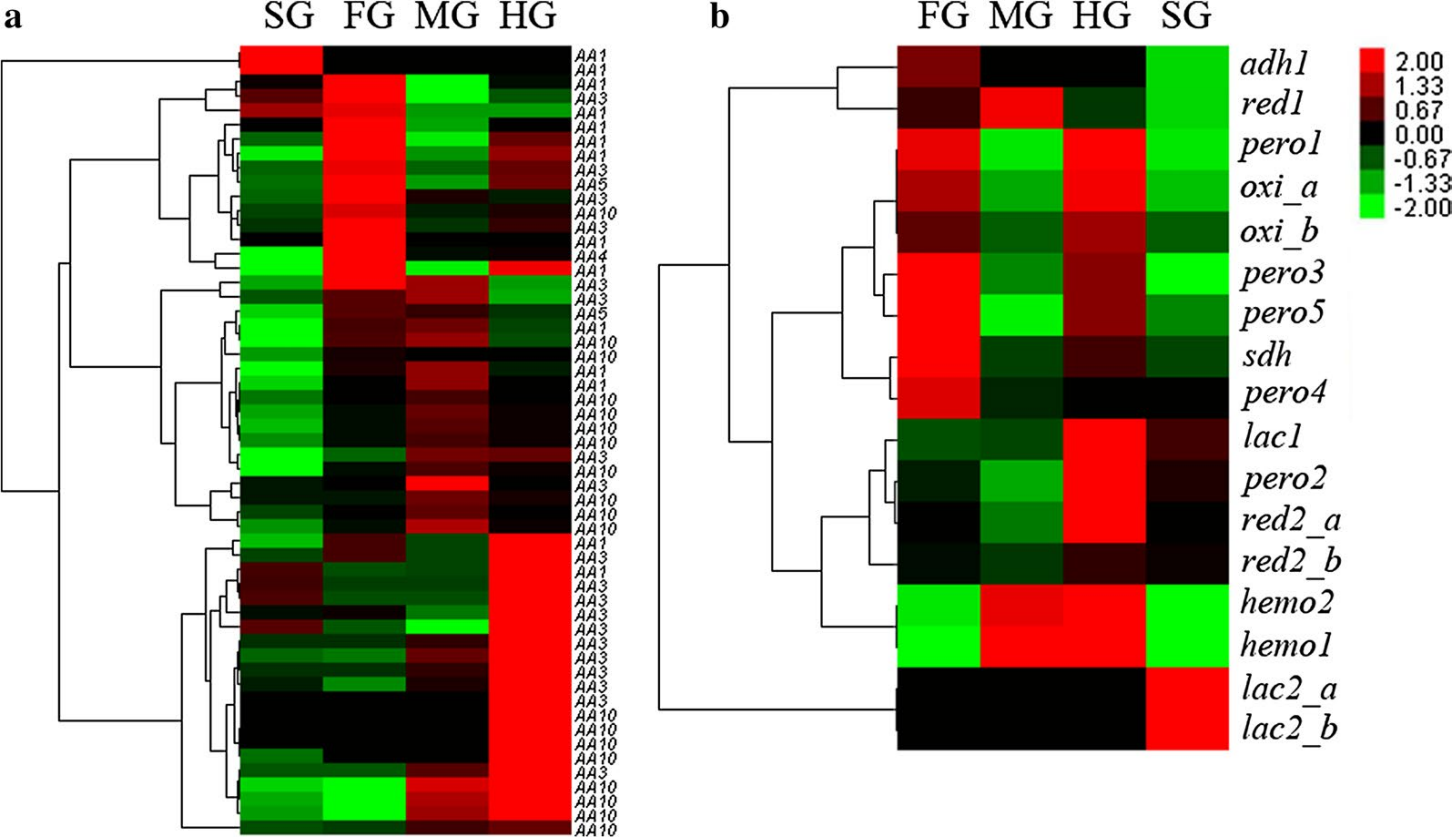
Clustering heatmap of redox enzymes (AA family) that act in conjunction with CAZymes (**a**) and extracellular secreted redox enzymes (**b**). Each column represents one sample and each row represents one unigene. Expression value was log (*n* + 1) transformed

Apart from the carbohydrate degradation, the modification and separation of lignin is also an important step for efficient biomass degradation. Unlike the carbohydrate degradation, which is primarily catalyzed by CAZymes, the biological degradation, or modification of lignin, mainly depends on the auxiliary redox enzymes [13, 26]. By cluster analysis, the auxiliary redox enzymes that matched the auxiliary activities families (AA) from the CAZy database were identified in each of the four gut tissues (Fig. 5a, Additional file 7: Table S4), where the salivary glands seemed to express less redox genes than those of other three tissues. Among those redox genes, only 6 out of the 55 genes were putatively identified from protists, indicating that most of the redox enzymes were from termite host, rather than the gut symbiotic protists. Specifically, most of these redox genes were encoded for AA1, AA3, and AA10 enzymes.

### Secreted redox enzymes and their potential functions in lignin modification

Extracellular secreted redox enzymes were very likely involved in a lignin modification processing due to their easy access and contact to the solid biomass substrates. Three out of the aforementioned 55 redox genes (Additional file 7: Table S4) were identified to encode extracellular proteins. As a supplement, additional extracellular secreted redox genes were also identified in our transcriptome by using BLAST against the newly found redox enzymes in another wood-feeding termite, *R. flavipes* [21], and by prediction of the sub-cellular localizations of the corresponding proteins. A total of 14 genes (17 unigenes) encoding extracellular secreted redox enzymes were identified from our transcriptome data (Table 1). Each of the three genes (*oxi*, *lac2*, and *red2*) had two alternative splicing products (unigenes).

**Table 1:** List of 14 predicted secreted redox enzymes

Gene name	Unigene name	BLASTx best hits		
		Accession no.	Annotation (organism)	*E*-value
*adh1*	Unigene9072_All	XP_021913051.1	Aldehyde dehydrogenase (*Zootermopsis nevadensis*)	5.8E−71
*lac1*	Unigene52482_All	AON96381.1	Laccase 1 (*Coptotermes formosanus*)	0
*pero1*	Unigene7661_All	XP_021933007.1	Peroxidasin homolog (*Zootermopsis nevadensis*)	7.4E−85
*pero2*	Unigene14041_All	XP_021936525.1	Glutathione peroxidase (*Zootermopsis nevadensis*)	1.0E−86
*red1*	Unigene14679_All	XP_021924937.1	7-dehydrocholesterol reductase, putative (*Zootermopsis nevadensis*)	3.3E−103
*oxi*	CL1400.Contig1_All (*oxi_a*)	XP_021929859.1	Lysyl oxidase homolog 2 (*Zootermopsis nevadensis*)	0
	CL1400.Contig2_All (*oxi_b*)			
*hemo2*	Unigene16668_All	AAU20852.2	Hexamerin II (*Reticulitermes flavipes*)	0
*sdh*	Unigene32098_All	XP_021933042.1	Epidermal retinol dehydrogenase 2-like (*Zootermopsis nevadensis*)	6.2E−65
*pero3*	Unigene22691_All	XP_021921010.1	Peroxidase-like (*Zootermopsis nevadensis*)	0
*pero4*	Unigene27848_All	AIP87047.1	Peroxidase precursor (*Macrotermes barneyi*)	6.00E−11
*lac2*	CL3975.Contig1_All (*lac2_a*)	ACX54558.1	Laccase 6 (*Reticulitermes flavipes*)	0
	CL3975.Contig2_All (*lac2_b*)			
*pero5*	Unigene2776_All	AIP87047.1	Peroxidase precursor (*Macrotermes barneyi*)	0
*red2*	CL2997.Contig1_All (*red2_a*)	XP_021924147.1	Gamma-interferon-inducible lysosomal thiol reductase (*Zootermopsis nevadensis*)	4.6E−100
	CL2997.Contig2_All (*red2_b*)			
*hemo1*	Unigene8398_All	AIO11839.1	Hemocyanin-like protein (*Coptotermes formosanus*)	0

The 17 unigenes that encode extracellular secreted redox enzymes were commonly assigned into the Isoptera order; thus, they were very likely produced from the termite host origin, rather than the gut symbionts. Interestingly, most of these unigenes were also tissue-specifically expressed (Fig. 5b). Two transcripts of the laccase gene, *lac2*, were enriched in the salivary glands. Five genes, *adh1*, *sdh*, *pero3*, *pero4*, and *pero5*, were enriched in the foregut. One gene, *red1*, was highly expressed in the midgut. Seven genes—*lac1*, *pero1*, *pero2*, two transcripts of *oxi*, *hemo1*, and *hemo2*—and one transcript of *red2* (*red2_a*) were enriched in the hindgut. Clearly, the characteristics of the tissue-specific expression of these genes suggest a successive reaction for biomass deconstruction with the corresponding enzymes in different parts of the gut system.

### Expression analyses of auxiliary redox genes in response to feeding on different biomass diets

**Fig. 6:**
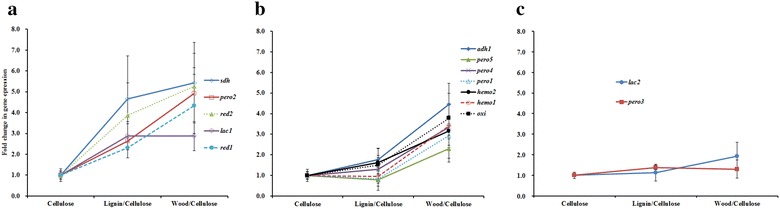
Quantitative RT-PCR analysis of expression of the 14 redox genes in responses to different diets in termite gut system. Termites were fed with three different food types: avicel cellulose, lignin alkali, and wood. The information of genes from group 1 (**a**), group 2 (**b**), and group 3 (**c**) was as listed in Table 1. A β-actin gene, *actin*, (CL2921.Contig1_All and CL2921.Contig2_All) was set as the reference gene

In order to further reveal the role of secreted redox enzymes in lignin modification, the termite workers were fed for 20 days with three types of diets: cellulose, lignin, and wood sawdust. Transcriptional profile of 14 genes coding secreted redox enzymes in response to different diets was obtained using quantitative RT-PCR. Based on gene expression profiling, the genes can be classified into 3 groups. In the first group, five genes were significantly upregulated (*P* < 0.05), both in the lignin-diet treatment and the wood-diet treatment (*sdh*, *pero2*, *red2*, *lac1*, and *red1*), as compared to those in the cellulose-diet treatment (Fig. 6a). In the second group, seven genes were significantly induced (*P* < 0.05), but only in the wood-diet treatment, as opposed to those in the cellulose-diet treatment (*adh1*, *pero1*, *pero4*, *pero5*, *oxi*, *hemo1*, and *hemo2*), while the gene expression levels were not significantly different between those in the lignin-diet and those in the cellulose-diet treatments (Fig. 6b). In the third group, the expression levels for each gene were not significantly different among the above-mentioned three treatments (*lac2* and *pero3*) (Fig. 6c). Generally, in the first and second group, auxiliary redox genes were almost all expressed at higher levels in the wood-diet treatment than those in the lignin-diet treatment, suggesting that wood was a more effective inducer than lignin for the redox genes. The gene expression pattern indicated that termite gut could regulate redox enzymes to respond to different biomass compositions. Apparently, the wood-induced auxiliary redox genes (group 1 and 2) were dispersedly expressed in different gut tissues (Fig. 5b), suggesting the lignin modification process was successive through the whole gut system.

## Discussion

### Coordination between carbohydrate-active enzymes from host and those from symbionts

Both the diversity and abundance analysis for the CAZymes indicated that host and symbiont complement their biocatalytic system to achieve effective biomass deconstruction (Fig. 3 and Additional file 6: Table S3). In fact, the importance of GH9 enzyme from the termite host has been verified by a previous study, where RNA interference of a homologous gene of GH9 (*cell*-*1*) for 24 days led to significant decrease in endoglucanase activity and significant mortality in termite *Reticulitermes flavipes* [27]. While abundant GH7, GH5, and GH11 mRNAs have been identified in other termite libraries, primarily from the symbiont [17, 21], suggesting their prevalent and essential roles in lower wood-feeding termites. The diverse GH7, GH5, and GH11 protistan enzymes might play an important role in complementing the termite GH enzymes for efficient biomass degradation. Overall, our results revealed the complementary function of GH enzymes between host and protists, where the host and protists produce different types of enzymes for biomass degradation. Considering that protists are primarily resided in the hindgut, the differential enzyme production also contributed to the different biomass degradation capacities in various gut organs.

### Secreted auxiliary redox enzymes might be responsible for the lignin modification

Termite host might be in charge of lignin modification for better utilization of woody biomass. Most of the AA enzymes, including the newly identified secreted redox enzymes, were produced from termite host, rather than the gut symbiotic protists (Additional file 7: Table S4 and Fig. 5), which was in consistence with the situation in *R. flavipes* [21]. These enzymes can be accounted for those candidate enzymes for lignin modification. For the identified AA1, AA3, and AA10 enzymes, it is known that most of the AA1 enzymes are multicopper oxidases that use diphenol and related substances as donors with oxygen as the acceptor [13]. The AA3 enzymes belong to the glucose-methanol-choline oxidoreductase family that contains a FAD-binding domain. The AA10 enzymes are lytic polysaccharide monooxygenases towards chitin or cellulose [13], and are cellulolytic redox enzymes rather than lignin-degrading enzymes. Specifically, a homogenous AA1 laccase from termite *Reticulitermes flavipes* has been proven to modify lignin alkali [14], and such AA1 laccases were deduced to be a kind of biological pretreatment enzymes. In our previous work, Hemo1 [28] and recombinant Lac1 [15], both from *C. formosanus*, have been proved to oxidize ABTS [2,2′-Azino-bis (3-ethylbenzothiazoline-6-sulfonic acid)] or hydroquinone, respectively, and they were very likely involved in lignin modification in termite gut system.

In particular, the programmatic redox-based lignin modification involves laccase- and peroxidase-based reactions throughout the whole gut. The result is consistent with the previous study that the oxygen levels in the different gut sections of *C. formosanus* were very different [29]. The tissue-specific expression of these redox genes was probably relevant with the levels of the potential oxiders (including oxygen) presented in its gut system.

Lignin-induced redox enzyme genes were very likely involved in lignin modification. As a matter of fact, diet-induced differential gene expression was also observed in other termite species [30] as well as some insects [31]. Raychoudhury et al. reported that the wood diet induced 253 ESTs in a wood-feeding termite species, *R. flavipes*. Among these ESTs, 82% are from the termite host. However, when feeding on cellulose, 88% of 293 enriched ESTs were from symbionts [30]. These results well correlate with our study in which peroxidase and alcohol-dehydrogenase genes were truly induced by wood and lignin substrates (Fig. 6). These results indicated an essential role of these auxiliary redox enzymes in modifying lignin structure. Further studies are needed to investigate how these enzymes synergize each other to modify the lignin structure in a termite gut system.

### The relative roles of termite host and its symbiotic protists in a dual digestive system

Regardless of the contribution of symbiotic prokaryotes, our transcriptome data analysis has implied that termite host may play a predominant role in biomass degradation, although its digestive mechanism has been approved to be synergistic between termite host and its eukaryotic symbionts (protists). In this so-called dual system processing, the roles of host termite and its gut symbionts, in particular, various flagellates (protists), have always been controversial. The earlier investigations for a lower wood-feeding termite, *R. flavipes*, suggested that the gut symbiotic protists played an essential role in biomass degradation due to its high percentage of CAZy genes (66% cellulase and 69% hemicellulase genes) identified from symbiotic protists rather than the termite host [21], which was primarily concluded from two relative small cDNA libraries, a total of 6555 unigenes found. However, our high-throughput data suggested that the termite host, *C. formosanus*, possessed a relatively complete enzyme system. The host transcriptome is more diverse and abundant as opposed to its symbiotic protists in terms of CAZymes and auxiliary redox enzymes.

This study also revealed that the lignin and carbohydrate degradation is a continuous and integrated process, where different groups of CAZymes and redox enzymes were successively and programmatically expressed in different organs of the termite gut system. First, the enzymes are complementary between the host and the symbionts. Most of the highly expressed GH, GT, CE, and CBM genes were from termite hosts, while some GH7, GH5, GH3, and CBM37 genes were primarily originated from gut protists. In addition, most of the redox enzymes involved in lignin modification processing was also confirmed from termite host, especially the secreted redox enzymes. Second, the organ-specific expression of different types of enzymes constituted a continuous reaction system that degrades biomass substrates efficiently using different chambers of its gut system. Third, termite gut is a highly dynamic system responding to different types of biomass. The expressions of several secretive enzymes during the feeding assay further revealed that twelve essential redox enzyme genes from termite host (*sdh*, *pero2*, *red2*, *lac1*, *red1*, *adh1*, *pero1*, *pero4*, *pero5*, *hemo1*, and *hemo2*) are promptly responsive to the wood-diet treatment, suggesting that termite itself can adjust its enzymatic system in respond to various biomass diets.

### Deduction of the biomass degradation mechanisms in *C. formosanus*

**Fig. 7:**
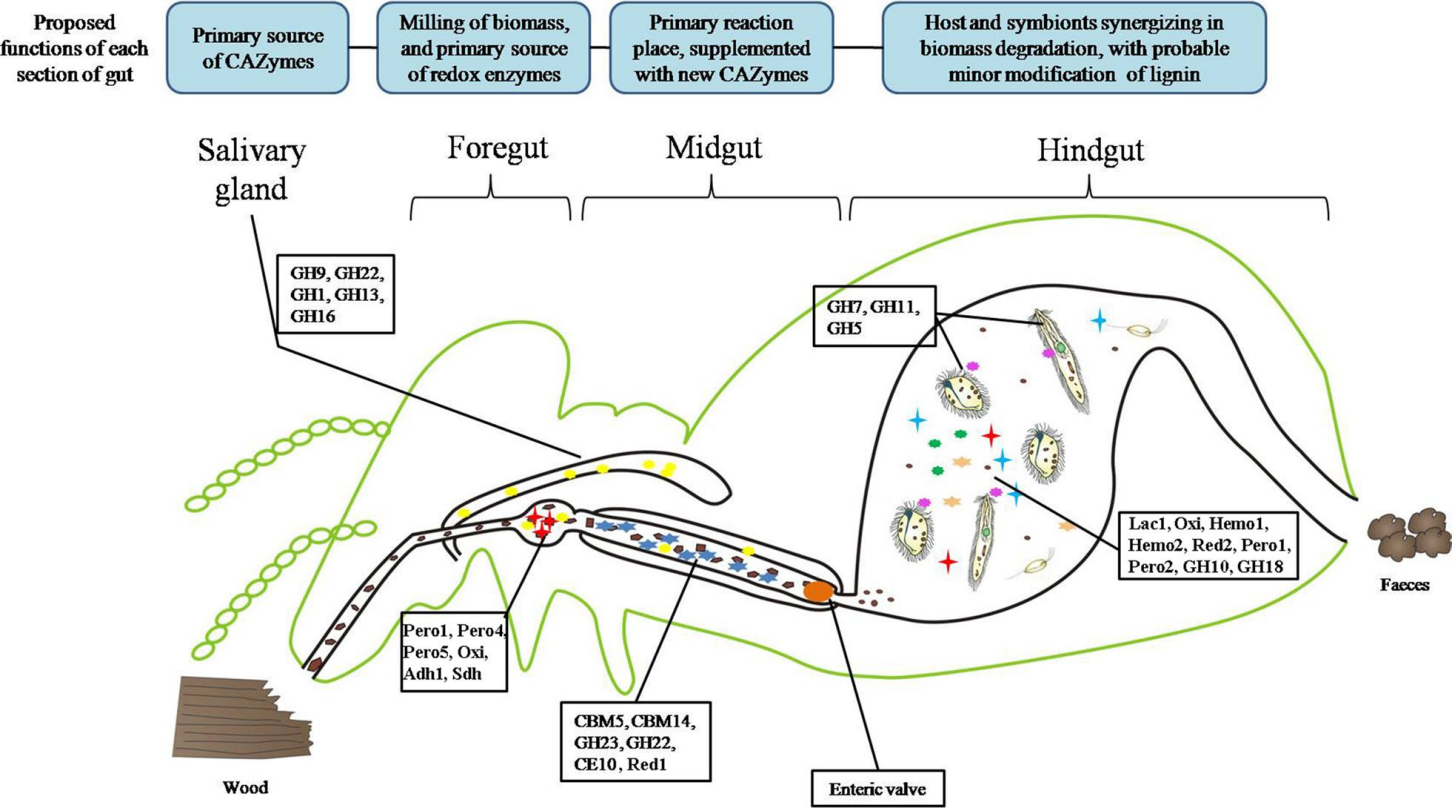
A proposed processing model (mechanisms) for lignocellulose degradation by wood-feeding termite, *Coptotermes formosanus*, where an array of carbohydrate and redox enzymes presented in the salivary gland, foregut, midgut, and hindgut were identified by transcriptome data analysis. This unique mechanism has suggested an integrative and synergistic system function for various gut compartments during the degradation processing on lignocellulosic biomass. Enteric valve could inhibit the passage of up-flow enzymes to the hindgut and probably protects the oxidative reaction condition in the midgut

The biomass degradation in termite digestive systems depends on some essential redox enzymes presented in foregut and midgut, as well as an array of the CAZymes across the whole gut system, from salivary glands, fore-, mid-, and hindgut. These enzymes were successively coordinated between the termite host and its symbiotic protists. Based on the transcriptomics data, we derived a putative mechanism (as shown in Fig. 7) to illustrate the biocatalytic networks in each gut tissue and their relationship, which suggested an integrative and synergistic system function during the biomass degradation processing. Once a tiny piece of woody biomass was chewed by the termite mandibles, the foregut gizzard (muscular proventriculus) began to mill it and to secrete peroxidases along with some peroxidases and oxidases (e.g., Pero1, Pero4, Pero5, Oxi, Adh1, and Sdh) to modify the lignin components for biomass pretreatment. Simultaneously, large amount of cellulase such as GH9, GH22, GH1, GH13, and GH16 from the salivary glands were secreted into the foregut and midgut to initiate the primary hydrolysis. Subsequently, in the midgut, fine wood particles were further hydrolyzed and modified, with additional CAZymes and auxiliary redox enzymes involved, such as CBM5, CBM14, GH23, GH22, CE10, Red1, Hemo1, and Hemo2. Although there were only quite a few redox enzymes expressed in the midgut, the former redox enzymes from the foregut could move to the down-flow midgut along with the substrates and allow adequate modification on lignin. Especially, there is an enteric valve between midgut and hindgut, which inhibits the passage of up-flow enzymes to the hindgut [32] and protects the oxidative reaction condition in the midgut. Finally, in the hindgut, some wood particles would be endocytosed by protists [33] and further hydrolyzed (mainly by GH7, GH11, and GH5 enzymes), while others would be further hydrolyzed by the above-mentioned GHs and hindgut-secreted GH10, GH18, etc. Notably, further lignin modification might persist in the hindgut, because the seven putative redox genes, *lac1*, *oxi*, *red2*, *hemo1*, *hemo2*, *pero1*, and *pero2* were enriched in the hindgut (Fig. 5b), where oxygen does exist in the place near the intestinal wall as an oxidant [29]. This continuous coordinative lignin modification and carbohydrate hydrolysis ensured the rapid consumption of cellulose and hemicellulose in the woody biomass. The coordination of oxidative enzymes and hydrolysis enzymes are very different from the current biorefinery schema [34] and should be considered in the future bioprocess design.

### Advantages of termite digestive systems for biomass degradation processing

As aforementioned, *C. formosanus* can rapidly accomplish its biomass degradation process within 24 h with a unique strategy that partially degrades and modifies lignin components (25–30%) [4, 5, 8], to maximize the carbohydrate utilization from biomass [5, 6], instead of with a time-consuming strategy for weeks or months by fully degradation of lignin components in biomass, such as a typical lignin processing from some white rot fungi or bacteria. The transcriptomic data analysis has indicated some differences in this regard from *C. formosanus* to certain extent. First, the termite digestive system had less lignin-degrading enzymes than white rot fungi that were reported with MnP, VP, LP, laccase, and many accessory enzymes for the generation of various radicals to attack the structure of lignin polymers [26, 35]. For example, a white rot fungus, *Phanerochaete chrysosporium* has been reported with 16 secreted peroxidases [26], while in *C. formosanus*, there were only 5 peroxidases detected in its digestive system. It is rational that a termite digestive system would not tolerate too much radical generation that may be harmful. Hence, it has been proposed recently that the auxiliary redox enzymes in *C. formosanus* mainly played a role in lignin structure modification, instead of a full deconstruction of lignin components in a wood diet. *C. formosanus* would leave lignin polymers in a relatively intact structure and further discharge them in its feces [22] because the ful digestion of lignin polymers possibly resulted in many intermediate products with more energy and time inputs, which might also negatively induce an inhibition effect on the CAZymes presented in its gut system [36].

Second, termite digestive system seems to be a continuous processing system, where GH and oxidative enzymes synergize the biomass degradation processing by a unique collaboration mechanism between termite host and its gut symbionts. Thus, the expression of CAZymes and auxiliary redox enzymes in different compartments of termite digestive system should be successively maintained and coordinated between termite host and its symbionts to ensure a unique composition of hydrolysis and oxidative enzymes. The outcomes of this synergistic reaction would allow the cellulose and hemicellulose utilization at an efficient mode with minimized energy inputs and time consumes to obtain enough nutrients from biomass. The mechanism of synergized biomass deconstruction in termite digestive system is suggested to be very helpful to develop a nature-inspired technology or strategy in guiding the modern biorefinery design, which may possibly pave a way for future breakthroughs and innovations in associated areas of industrial biotechnology [5, 6, 37].

## Conclusions

The wood-feeding termite system of *C. formosanus* has been proposed as a dual digestive system in biomass degradation processing co-functioned by termite host and its gut symbionts, where termite host was found to be predominant over symbiotic protists in terms of both gene diversity and its transcript abundance when focused on both CAZyme genes and auxiliary redox genes detected with transcriptomics analysis. The compartmented/tissue-based expression of different kinds of enzymes in termite digestive system allowed a continuous and sequenced degradation processing, where CAZymes and auxiliary redox enzymes synergized a series of reactions on biomass deconstruction. More importantly, twelve genes that encode extracellular secreted redox enzymes showed some rapid responses towards a wood-rich diet feeding, and very likely, these redox enzymes might be crucial for the lignin modification processing. It seems that the foregut is a very important place for lignin modification processing due to 6 out of the 12 redox genes being expressed from there, and further, the lignin modification processing might be continuously maintained in the hindgut because four redox genes were specifically expressed there. However, salivary glands seemed to secret large amount of GHs and initiate the primary hydrolysis, and midgut seemed to serve as an inevitable reaction location to continue the hydrolysis and modification of biomass using enzymes that were mainly secreted from up-flow organs (saliva glands and foregut). These findings demonstrate that the termite digestive system of *C. formosanus* was a unique biomass degrading system evolved with salivary glands, foregut, midgut, and hindgut for different functions that fully reflected an integrative and synergistic mechanism between termite host and its gut symbionts in biomass degradation processing.

## Methods

### Termite collection and dissection

The workers of wood-feeding termite, *C. formosanus*, were originally collected from Hangzhou city, Zhejiang province, P. R. China, and were then maintained in the laboratory by feeding on pine wood, *Pinus massoniana* Lamb at 25 ± 2 °C. Thousands of termite workers, in 3–4 instar from same colony, were carefully dissected under a dissect microscopy, and their tissues of SG, FG, MG, and HG from a termite worker gut system were individually collected and preserved in RNAlater (Qiagen, Valencia, CA) at − 80 °C until use. Termite gut structure is an elongated tube differentiated into the foregut, midgut, and hindgut, where the foregut with the esophagus, crop, and gizzard (muscular proventriculus), the midgut with a simple tube of uniform diameter distally inserted by some Malpighian tubules, and at the end, a highly developed hindgut mainly harboring various symbiotic microbes, including various flagellates (cellulolytic protists) (Additional file 1: Figure S1). In addition, the saliva glands linked to the foregut with paired gland tissues in the termite gut system are primarily responsible to produce an array of enzymes involved in biomass degradation processing.

### RNA extraction and sequencing

Total RNA was isolated from four individual parts of a digestive organ of termite workers (SG, FG, MG, and HG) using the Axygen RNA extraction Kit (Axygen, Hangzhou, China) according to the manufacture’s protocol. The RNA was treated with RNase-Free DNase set (QIAGEN Inc., Valencia, CA, USA) to digest any genomic DNA that might be present. Total RNA was quantified using an ultraviolet (UV) spectrophotometer and the RNA quality and integrity were examined using an RNA Lab-On-A-Chip (Caliper Technologies Corp., Mountain View, CA, USA), which was evaluated on an Agilent Bioanalyzer 2100 (Agilent Technologies, Palo Alto, CA, USA). Total RNA was sent to the Beijing Genomics Institute (BGI, Shenzhen, China) for library preparation and sequencing. PolyA (+) mRNAs were enriched using the Oligo (dT) 25 Magnetic Beads. Then the mRNAs were fragmented, followed by the synthesis of cDNA using random hexamer-primer for the first-strand cDNA and buffer, dNTPs, RNase H and DNA polymerase I for the second-strand cDNA synthesis. The cDNA fragments were then purified and connected with sequencing adapters. Fragments with a length of 200 bp were selected by agarose gel electrophoresis and sequenced on the HiSeq2000 platform (Illumina, Inc., San Diego, CA, USA). The raw sequencing reads were deposited in Short Read Archive at NCBI under the accession numbers from SRR2155575 to SRR2155578.

### Transcriptome assembly

Raw reads were filtered using an in-house perl script to remove reads with a sequencing adaptor and reads with low sequencing quality (either reads with more than 20% of bases with a quality score of *Q* ≤ 10 or percentages of ambiguous sequences, “*N*” > 5%). Clean paired-end reads were then subjected to the assembly process. Trinity software [38] was used for transcript construction. Reads from each library were assembled separately first, and then we used the sequence clustering tool TGICL [39] to cluster those transcript to reduce the sequence redundancy and get the unigene sequences that we used for further analysis.

### Sequence annotation and taxonomy analysis

Unigenes were annotated by searching against the Nr (non-redundant protein) and Nt (nucleotide) database of NCBI, as well as the Swiss-Prot database, COG database, and KEGG database, using BLASTx with *E*-value cutoff of 1E−5. Based on the BLASTx search result, the CDS of the unigenes were identified through their homologous proteins. For those unigenes without homologous proteins, ESTScan [40] was used to predict the ORF, where the longest ORFs were taken as the CDS region of the unigenes. CDS sequences were then translated to protein sequences in silico. Annotations for the GO were obtained by using the Blast2go package [41] based on the homologous protein sequences that were identified from the Nr database. All the names of species were also obtained from the Nr annotation. According to the Taxonomy database of NCBI, the full taxonomy tree of the species for the unigenes was obtained. Unigenes which best matched (according to BLAST search *E*-value) homologous proteins from the Animalia group were taken as the termite genes, and unigenes which best matched homologous proteins from the Parabasalia group were taken as genes from the symbiotic protists.

### Analysis of CAZymes and auxiliary redox enzymes

All the assembled unigenes were submitted for local BLASTx search against the CAZy database (http://www.cazy.org), with an *E*-value cutoff of 1E−5 to filter the BLAST search results. The species’ origins of CAZymes and auxiliary redox enzymes were determined by considering both the specie names of homologous proteins in the Nr database and the CAZy database. Sequences of the newly identified CAZymes and redox enzymes were submitted to the SignalP prediction server (http://www.cbs.dtu.dk/services/SignalP/) to perform the signal peptide prediction, using the parameters of eukaryotic organism group and default D-value cutoffs. In order to identify genes that encode for extracellular secreted enzymes, sub-cellular localization of the corresponding proteins was predicted by the ProtComp 9.0 program (for Animals and Fungi, http://www.softberry.com).

### Gene expression quantification

To quantify the expression of unigenes, reads were mapped to the unigene sequence using a SOAP aligner allowing a maximum of three mismatches in one read [42]. An in-house Perl script was used to count the number of reads that mapped to each unigene. Only the reads that uniquely mapped to one unigene were counted, while the reads that ambiguously mapped to more than one unigenes were discarded. Fragment (one pair end) counts were then normalized according to the definition of FPKM value, and the latter was used to represent the comparable expression values among samples.

### Clustering analysis

To compare expression profiles, a hierarchical clustering analysis was carried out using Cluster v3.0 [43]. The expression values of all selected genes were normalized and transformed into logarithmic (base 2) values. A gene tree in the clustering was generated by calculating the Pearson Correlation and the Average linkage clustering method, and the results were visualized using TreeView v1.0.5 software [44].

### Feeding tests and quantitative RT-PCR detection

Three kinds of artificial diets for termites were made from 500 mg of Avicel Ph101 (Sigma, Shanghai, China), lignin alkali (Sigma 471003), or sawdust of wood *Pinus massoniana* Lamb, each supplemented in 20 ml 2% agar, according to Tanaka et al. [45] with minor modifications. After a boil-and-cool-down step, the solidified foods were then cut into about 5 mm × 5 mm × 50 mm blocks. Three groups of 30 termite workers were fed separately on these three kinds of food blocks for 20 days at 25 ± 2 °C. Termite workers, fed on different substrates, were then sacrificed for total RNA extraction of the whole bodies (2–4 dead termites out of 30 were excluded), using a multisource total RNA miniprep kit (Axygen) following the manufacturer’s instructions. Subsequently, the RNAs were reverse transcribed into cDNAs using the One-step genomic DNA removal and cDNA synthesis kit (TransGen, Beijing, China). Finally, the cDNAs were used as templates to conduct the quantitative RT-PCR, employing gene-specific primers as listed in Additional file 8: Table S5. The β-actin gene *actin* was set as the reference gene. Gene expression was quantified using the 2^−ΔΔCT^ Method [46]. Student’s *t* test employing a two-tailed test was used for significance analysis (Excel 2010, Microsoft, Seattle, WA, USA).

## Additional files

**Additional file 1: Figure S1.** Morphology and features of the gut system of a wood-feeding termite, *Coptotermes formosanus*: (A) The worker termite; (B) The gut system of a work termite under a dissect microscopy, where termite gut structure is an elongated tube differentiated into the foregut (FG), midgut (MG), and hindgut (HG), where the foregut with the esophagus, crop, and gizzard (muscular proventriculus), the midgut with a simple tube of uniform diameter distally inserted by some Malpighian tubules, and at the end, a highly developed hindgut mainly harboring various symbiotic microbes, including various flagellates (cellulolytic protists). In addition, the saliva glands (SG) linked to the foregut with paired gland tissues in termite gut system are primarily responsible to produce an array of enzymes involved in biomass degradation processing.

**Additional file 2: Table S1.** Transcriptome sequencing and assembly. Reads with low sequencing quality were removed and clean paired-end reads were subjected to the assembly process.

**Additional file 3: Table S2.** Annotation of unigenes. The BLAST results of the unigenes against the Nr, Nt, and Swissprot database were listed. The names of species were obtained from the Nr annotation.

**Additional file 4: Figure S2.** Percentages of unigenes that assigned as termite gene and protistan gene from the four termite digestive organs: salivary gland (SG), foregut (FG), midgut (MG) and hindgut (HG).

**Additional file 5: Figure S3.** Summary of percentages of CAZyme genes encoding for Glycoside Hydrolases (GH), Glycosyl Transferases (GT), Carbohydrate Esterases (CE) and Carbohydrate binding modules (CBM) from termite and its gut symbiotic protists.

**Additional file 6: Table S3.** Expression of CAZyme genes. All the assembled unigenes were submitted for local BLASTx search against the CAZy database (http://www.cazy.org), with an *E*-value cutoff of 1E−5. The specie origins of CAZymes were determined by considering both the specie names of homologous proteins in the Nr database and the CAZy database. The FPKM value of each CAZyme gene in different gut tissues was listed.

**Additional file 7: Table S4.** Expression of auxiliary redox enzymes from AA families. All the assembled unigenes were submitted for local BLASTx search against the CAZy database (http://www.cazy.org), with an *E*-value cutoff of 1E−5. The specie origins of redox enzymes were determined by considering both the specie names of homologous proteins in the Nr database and the CAZy database. The FPKM value of each redox gene in different gut tissues was listed.

**Additional file 8: Table S5.** Primers for quantitative RT-PCR. All the candidate genes were from Fig. 6.

## Abbreviations

NBUS: natural biomass utilization systems
SG: salivary glands
FG: foregut
MG: midgut
HG: hindgut
RT-PCR: reverse transcription polymerase chain reaction
GTs: glycosyltransferases
CEs: carbohydrate esterases
CBMs: carbohydrate binding domains
PLs: polysaccharide lyases
CAZyme: carbohydrate-active enzymes
MnP: manganese peroxidases
VP: Versatile Peroxidase
LP: lignin peroxidase
FAD: flavin adenine dinucleotide
cDNA: complementary deoxyribonucleic acid
RNA: ribonucleic acid
ESTs: expressed sequence tags
NCBI: the national center for biotechnology information
GO: gene ontology
COG: the database of clusters of orthologous groups of proteins
KEGG: the database of kyoto encyclopedia of genes and genomes
CDS: coding sequence
ORF: open reading frame
FPKM: fragments per kilobase of transcript, per million fragments sequenced
ABTS: [2,2′-Azino-bis (3-ethylbenzothiazoline-6-sulfonic acid)]
