# Supplementary figures and images for: Transcriptome analysis of the digestive system of a wood-feeding termite (Coptotermes formosanus) revealed a unique mechanism for effective biomass degradation
Source: Biotechnol Biofuels. 2018 Feb 3;11:24. doi: 10.1186/s13068-018-1015-1 (PMC5797411; doi:10.1186/s13068-018-1015-1)

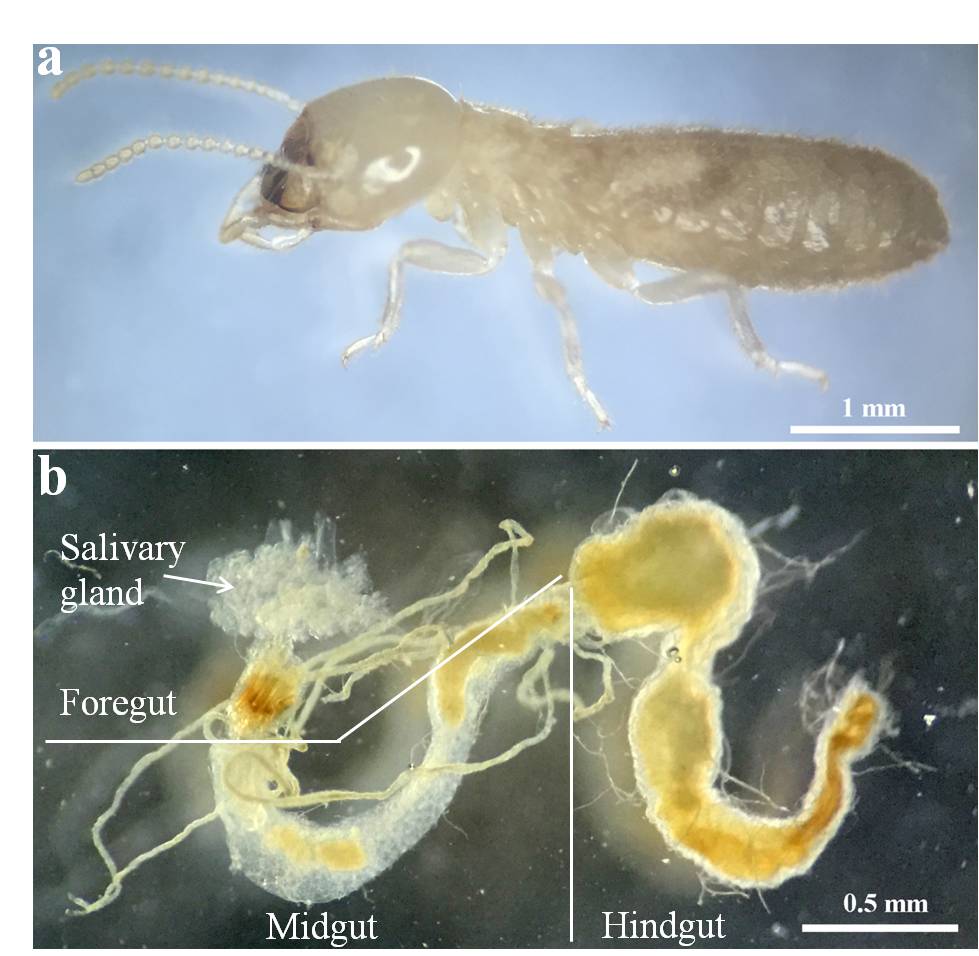

Supplement: Supplementary file 1 — Additional file 1: Figure S1. Morphology and features of the gut system of a wood-feeding termite, Coptotermes formosanus: (A) The worker termite; (B) The gut system of a work termite under a dissect microscopy, where termite gut structure is an elongated tube differentiated into the foregut (FG), midgut (MG), and hindgut (HG), where the foregut with the esophagus, crop, and gizzard (muscular proventriculus), the midgut with a simple tube of uniform diameter distally inserted by some Malpighian tubules, and at the end, a highly developed hindgut mainly harboring various symbiotic microbes, including various flagellates (cellulolytic protists). In addition, the saliva glands (SG) linked to the foregut with paired gland tissues in termite gut system are primarily responsible to produce an array of enzymes involved in biomass degradation processing. [file 13068_2018_1015_MOESM1_ESM.jpg]

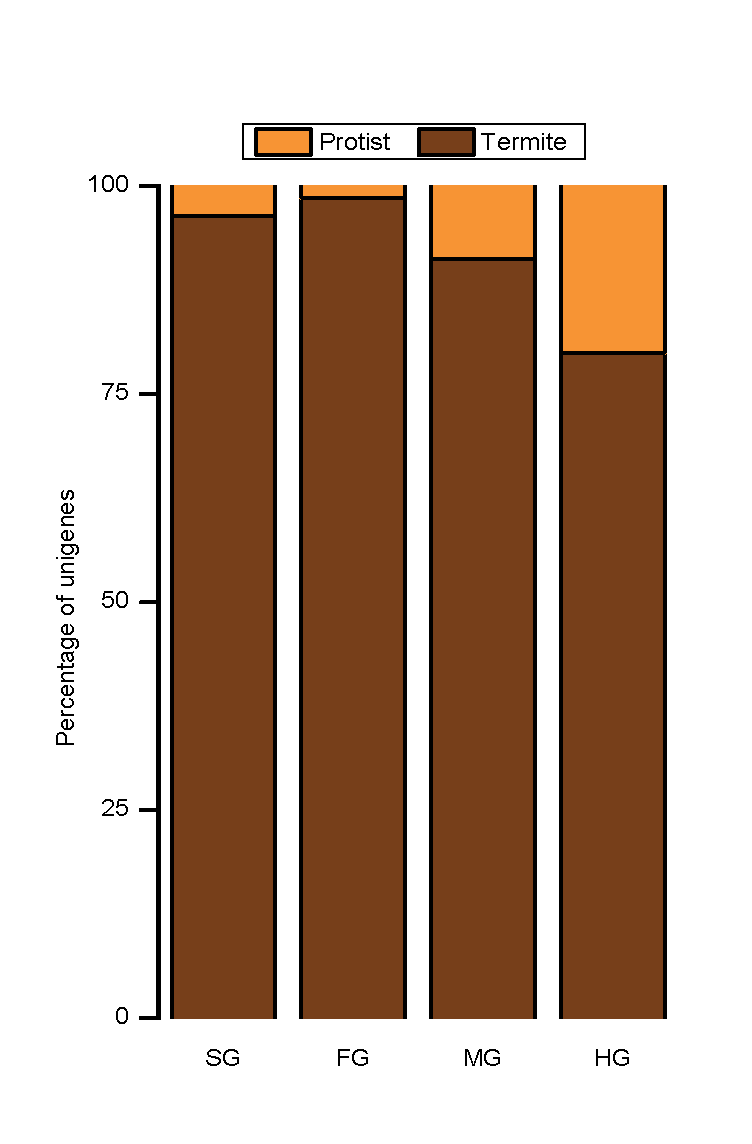

Supplement: Supplementary file 4 — Additional file 4: Figure S2. Percentages of unigenes that assigned as termite gene and protistan gene from the four termite digestive organs: salivary gland (SG), foregut (FG), midgut (MG) and hindgut (HG). [file 13068_2018_1015_MOESM4_ESM.tif]

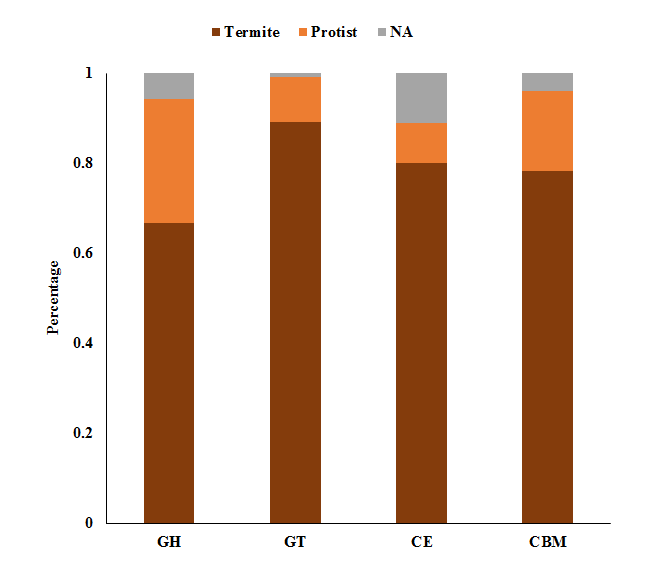

Supplement: Supplementary file 5 — Additional file 5: Figure S3. Summary of percentages of CAZyme genes encoding for Glycoside Hydrolases (GH), Glycosyl Transferases (GT), Carbohydrate Esterases (CE) and Carbohydrate binding modules (CBM) from termite and its gut symbiotic protists. [file 13068_2018_1015_MOESM5_ESM.tif]
