# Supplementary material for: Transcriptome analysis of the digestive system of a wood-feeding termite (Coptotermes formosanus) revealed a unique mechanism for effective biomass degradation
Source: Biotechnol Biofuels. 2018 Feb 3;11:24. doi: 10.1186/s13068-018-1015-1 (PMC5797411; doi:10.1186/s13068-018-1015-1)
Supplement: Supplementary file 2 — Additional file 2: Table S1. Transcriptome sequencing and assembly. Reads with low sequencing quality were removed and clean paired-end reads were subjected to the assembly process. [file 13068_2018_1015_MOESM2_ESM.doc]

**Table S1. Transcriptome sequencing and assembly.**

| Sample | Total reads | Total nucleotides | Assembled transcripts | Total transcript length | *N50* length of transcripts |
| --- | --- | --- | --- | --- | --- |
| Salivary glands | 13,599,086 | 1,161,084,060 | 15,195 | 6,341,728 | 423 |
| Foregut | 14,666,706 | 1,231,730,820 | 51,865 | 25,407,242 | 554 |
| Midgut | 14,353,012 | 1,192,424,760 | 44,544 | 19,821,315 | 541 |
| Hindgut | 14,949,160 | 1,240,601,040 | 57,687 | 23,385,429 | 456 |
| All | 57,567,964 | 4,825,840,680 | 71,117 | 37,989,847 | 648 |
